# ECDC’s latest publications

**DOI:** 10.2807/1560-7917.ES.2018.23.50.1812132

**Published:** 2018-12-13

**Authors:** Eurosurveillance editorial team

**Affiliations:** 1 European Centre for Disease Prevention and Control (ECDC), Stockholm, Sweden

**Keywords:** infectious diseases, public health, Europe

 

**Rapid risk and outbreak assessments**

 

Rapid risk assessment: Influenza-associated invasive pulmonary aspergillosis, Europe

Date of publication: 30 November 2018

https://ecdc.europa.eu/en/publications-data/rapid-risk-assessment-influenza-associated-invasive-pulmonary-aspergillosis

 

Rapid risk assessment: Multidrug-resistant *Staphylococcus epidermidis*

Date of publication: 8 November 2018

https://ecdc.europa.eu/en/publications-data/rapid-risk-assessment-multidrug-resistant-staphylococcus-epidermidis

 

Multi-country outbreak of *Listeria monocytogenes* sequence type 8 infections linked to consumption of salmon products

Date of publication: 25 October 2018

https://ecdc.europa.eu/en/publications-data/multi-country-outbreak-listeria-monocytogenes-sequence-type-8-infections-linked

 

Rapid risk assessment: Local transmission of dengue fever in France and Spain

Date of publication: 22 October 2018

https://ecdc.europa.eu/en/publications-data/rapid-risk-assessment-local-transmission-dengue-fever-france-and-spain

 

Rapid risk assessment: Ebola virus disease outbreak in North Kivu and Ituri Provinces, Democratic Republic of the Congo – first update

Date of publication: 5 October 2018

https://ecdc.europa.eu/en/publications-data/rapid-risk-assessment-ebola-virus-disease-outbreak-north-kivu-and-ituri-first-update

 

**Scientific advice**

****

Public health guidance in brief on HIV, hepatitis B and C testing in the EU/EEA

Date of publication: 5 December 2018

https://ecdc.europa.eu/en/publications-data/public-health-guidance-brief-hiv-hepatitis-b-and-c-testing-eueea

****

Public health guidance on screening and vaccination for infectious diseases in newly arrived migrants within the EU/EEA

Date of publication: 5 December 2018

https://ecdc.europa.eu/en/publications-data/public-health-guidance-screening-and-vaccination-infectious-diseases-newly

****

Public health guidance on HIV, hepatitis B and C testing in the EU/EEA

Date of publication: 23 November 2018

https://ecdc.europa.eu/en/publications-data/public-health-guidance-hiv-hepatitis-b-and-c-testing-eueea

****

Programmatic management of latent tuberculosis infection in the European Union

Date of publication: 15 October 2018

https://ecdc.europa.eu/en/publications-data/programmatic-management-latent-tuberculosis-infection-european-union

****

**Technical reports**

 

Hepatitis B and C testing strategies in healthcare and community settings in the EU/EEA

Date of publication: 11 December 2018

https://ecdc.europa.eu/en/publications-data/hepatitis-b-and-c-testing-strategies-healthcare-and-community-settings-eueea

 

Best practice recommendations for conducting after-action reviews to enhance public health preparedness

Date of publication: 5 December 2018

https://ecdc.europa.eu/en/publications-data/best-practice-recommendations-public-health-preparedness

 

Handbook on designing and implementing an immunisation information system

Date of publication: 20 November 2018

https://ecdc.europa.eu/en/publications-data/designing-and-implementing-immunisation-information-system-handbook

 

Retrospective surveillance and enhanced case-finding of congenital rubella syndrome cases

Date of publication: 16 November 2018

https://ecdc.europa.eu/en/publications-data/retrospective-surveillance-and-enhanced-case-finding-congenital-rubella-syndrome

 

The importance of vector abundance and seasonality

Date of publication: 12 November 2018

https://ecdc.europa.eu/en/publications-data/importance-vector-abundance-and-seasonality

 

External quality assessment of laboratory performance – European Antimicrobial Resistance Surveillance Network (EARS-Net), 2017

Date of publication: 12 November 2018

https://ecdc.europa.eu/en/publications-data/external-quality-assessment-laboratory-performance-european-antimicrobial

 

Laboratory procedures for diagnosis and typing of human *Clostridium difficile* infection

Date of publication: 19 October 2018

https://ecdc.europa.eu/en/publications-data/laboratory-procedures-diagnosis-and-typing-human-clostridium-difficile-infection

 

Eighth external quality assessment scheme for Salmonella typing

Date of publication: 15 October 2018

https://ecdc.europa.eu/en/publications-data/eighth-external-quality-assessment-scheme-salmonella-typing

 

Review of reviews and guidelines on target groups, diagnosis, treatment and programmatic issues for implementation of latent tuberculosis management

Date of publication: 15 October 2018

https://ecdc.europa.eu/en/publications-data/review-reviews-and-guidelines-target-groups-diagnosis-treatment-and-programmatic

 

**Surveillance reports**

 

Influenza virus characterisation, Summary Europe, October 2018

Date of publication: 11 December 2018

https://ecdc.europa.eu/en/publications-data/influenza-virus-characterisation-summary-europe-october-2018

 

HIV/AIDS surveillance in Europe 2018 - 2017 data

Date of publication: 28 November 2018

https://ecdc.europa.eu/en/publications-data/hivaids-surveillance-europe-2018-2017-data

 

Joint WHO/ECDC influenza virus characterisation report, summary of TESSy virus characterisation data 2017/18

Date of publication: 22 November 2018

https://ecdc.europa.eu/en/publications-data/influenza-virus-characterisation-report-summary-17-18

 

Surveillance of antimicrobial resistance in Europe 2017

Date of publication: 15 November 2018

https://ecdc.europa.eu/en/publications-data/surveillance-antimicrobial-resistance-europe-2017

 

Monthly measles and rubella monitoring report, November 2018

Date of publication: 9 November 2018

https://ecdc.europa.eu/en/publications-data/monthly-measles-and-rubella-monitoring-report-november-2018

 

Influenza virus characterisation, Summary Europe, September 2018

Date of publication: 30 October 2018

https://ecdc.europa.eu/en/publications-data/influenza-virus-characterisation-summary-europe-september-2018

 

Monthly measles and rubella monitoring report, October 2018

Date of publication: 12 October 2018

https://ecdc.europa.eu/en/publications-data/monthly-measles-and-rubella-monitoring-report-october-2018

 

**Annual Epidemiological Report series on communicable diseases in Europe**

****

Crimean–Congo haemorrhagic fever - Annual Epidemiological Report for 2016

Date of publication: 12 December 2018

https://ecdc.europa.eu/en/publications-data/crimean-congo-haemorrhagic-fever-annual-epidemiological-report-2016

 

Ebola and Marburg fevers - Annual Epidemiological Report for 2016

Date of publication: 12 December 2018

https://ecdc.europa.eu/en/publications-data/ebola-and-marburg-fevers-annual-epidemiological-report-2016

 

Cholera - Annual Epidemiological Report for 2016

Date of publication: 11 December 2018

https://ecdc.europa.eu/en/publications-data/cholera-annual-epidemiological-report-2016

 

Antimicrobial consumption - Annual Epidemiological Report for 2017

Date of publication: 15 November 2018

https://ecdc.europa.eu/en/publications-data/antimicrobial-consumption-annual-epidemiological-report-2017

 

Echinococcosis - Annual Epidemiological Report for 2016

Date of publication: 30 October 2018

https://ecdc.europa.eu/en/publications-data/echinococcosis-annual-epidemiological-report-2016

 

Seasonal influenza - Annual Epidemiological Report for 2017 - 2018

Date of publication: 22 October 2018

https://ecdc.europa.eu/en/publications-data/seasonal-influenza-annual-epidemiological-report-2017-18-season

 

Zika virus infection - Annual Epidemiological Report for 2016

Date of publication: 12 October 2018

https://ecdc.europa.eu/en/publications-data/zika-virus-infection-annual-epidemiological-report-2016

 

Giardiasis (lambliasis) - Annual Epidemiological Report for 2016 

Date of publication: 12 October 2018

https://ecdc.europa.eu/en/publications-data/giardiasis-lambliasis-annual-epidemiological-report-2016

 

Smallpox - Annual Epidemiological Report for 2016

Date of publication: 12 October 2018

https://ecdc.europa.eu/en/publications-data/smallpox-annual-epidemiological-report-2016

 

Lassa fever- Annual Epidemiological Report for 2016

Date of publication: 12 October 2018

https://ecdc.europa.eu/en/publications-data/lassa-fever-annual-epidemiological-report-2016

 

Rabies - Annual Epidemiological Report for 2016

Date of publication: 10 October 2018

https://ecdc.europa.eu/en/publications-data/rabies-annual-epidemiological-report-2016

 

Plague - Annual Epidemiological Report for 2016

Date of publication: 10 October 2018

https://ecdc.europa.eu/en/publications-data/plague-annual-epidemiological-report-2016

 

Hantavirus infection - Annual Epidemiological Report for 2016

Date of publication: 5 October 2018

https://ecdc.europa.eu/en/publications-data/hantavirus-infection-annual-epidemiological-report-2016

 

Yellow fever - Annual Epidemiological Report for 2016

Date of publication: 5 October 2018

https://ecdc.europa.eu/en/publications-data/yellow-fever-annual-epidemiological-report-2016

 

Chikungunya virus disease - Annual Epidemiological Report for 2016

Date of publication: 1 October 2018

https://ecdc.europa.eu/en/publications-data/chikungunya-virus-disease-annual-epidemiological-report-2016

 

**Mission reports**

 

ECDC country visit to Malta to discuss antimicrobial resistance issues, 3-7 July 2017

Date of publication: 15 November 2018

https://ecdc.europa.eu/en/publications-data/ecdc-country-visit-malta-discuss-antimicrobial-resistance-issues-3-7-july-2017

 

**Corporate publications**

****

Long-term Surveillance Strategy 2014-2020 (revised)

Date of publication: 5 October 2018

https://ecdc.europa.eu/en/publications-data/long-term-surveillance-strategy-2014-2020-revised

 

**ECDC communicable disease threats report**

Date of publication: every Friday

https://ecdc.europa.eu/en/threats-and-outbreaks/reports-and-data/weekly-threats

**

**Scientific peer-reviewed publications**

Publications by ECDC staff in scientific journals

Updated monthly

https://ecdc.europa.eu/en/publications-data?f%5b0%5d=output_types%3A1247

